# FIT for purpose: study protocol for a randomized controlled trial to personalize surveillance colonoscopy for individuals at elevated risk of colorectal cancer

**DOI:** 10.1007/s00384-023-04493-8

**Published:** 2023-07-25

**Authors:** Jean M. Winter, Kathryn J. Cornthwaite, Graeme P. Young, Carlene Wilson, Gang Chen, Richard Woodman, Michelle Coats, Robert Fraser, Charles Cock, Peter Bampton, Erin L. Symonds

**Affiliations:** 1 Cancer Research, College of Medicine and Public Health, Flinders University, Bedford Park, SA Australia; 2 Melbourne School of Population and Global Health, University of Melbourne, Parkville, VIC Australia; 3 Centre for Health Economics, Monash University, Caulfield East, VIC, Australia; 4 Department of Gastroenterology & Hepatology, Flinders Medical Centre, Bedford Park, SA Australia

**Keywords:** Colorectal cancer, FIT, Colonoscopy surveillance, Fecal hemoglobin

## Abstract

**Purpose:**

There is increasing demand for colorectal cancer (CRC) surveillance, but healthcare capacity is limited. The burden on colonoscopy resources could be reduced by personalizing surveillance frequency using the fecal immunochemical test (FIT). This study will determine the safety, cost-effectiveness, and patient acceptance of using FIT to extend surveillance colonoscopy intervals for individuals at elevated risk of CRC.

**Methods:**

This multicenter, prospective, randomized controlled trial will invite participants who are scheduled for surveillance colonoscopy (due to a personal history of adenomas or a family history of CRC) and who have returned a low fecal hemoglobin (< 2 μg Hb/g feces; F-Hb) using a two-sample FIT (OC Sensor, Eiken Chemical Company) in the prior 3 years. A total of 1344 individuals will be randomized to either surveillance colonoscopy as scheduled or delayed by 1 or 2 years for individuals originally recommended a 3- or 5-year surveillance interval, respectively. The primary endpoint is incidence of advanced neoplasia (advanced adenoma and/or CRC). Secondary endpoints include cost-effectiveness and consumer acceptability of extending surveillance intervals, determined using surveys and discrete choice experiments.

**Conclusion:**

This study will establish the safety, cost-effectiveness, and acceptability of utilizing a low FIT Hb result to extend colonoscopy surveillance intervals in a cohort at elevated risk for CRC. This personalized approach to CRC surveillance will lead to a reduction in unnecessary colonoscopies, increases in healthcare savings, and a better patient experience.

**Trial registration:**

Registration was approved on December 9, 2019 with the Australian New Zealand Clinical Trials Registry ANZCTR 12619001743156.

## Introduction

Colorectal cancer (CRC) is the third most common non-cutaneous solid cancer worldwide accounting for 1.9 million newly diagnosed cases in 2020 and is the second leading cause of cancer related death, with almost 1 million deaths reported in 2020 [1]. These numbers are only set to rise, with an estimated 3.2 million cases of CRC globally in the year 2040 [2]. In the USA alone, CRC is estimated to rise from approximately 150–160,000 cases in 2020 to between 180 and 210,000 cases by 2040 [2, 3]. It has been well established that early detection and removal of colorectal neoplasia, including CRC and pre-cursor lesions called adenomatous polyps (adenomas), can prevent many deaths [4], and colonoscopy is considered the gold standard for detection and/or removal of such lesions. Following the diagnosis of colorectal neoplasia, ongoing regular surveillance colonoscopy is recommended. However, with expected large increases in the rates of CRC diagnoses, the workload demand on hospitals to perform the associated increase in surveillance colonoscopies will only become more cumbersome in what are already strained health care systems [5]. Additionally, although uncommon, the procedure carries an element of risk to the patient, with adverse events from anesthesia, bleeding or perforation [6]. Most importantly, over 85% of individuals at elevated risk of CRC undergoing surveillance colonoscopy due to prior neoplasia or a family history of CRC have no significant findings [7], demonstrating the potential overutilization of colonoscopy in this population. There is clearly a need to identify those individuals who do not need such intensive colonoscopy surveillance.

Screening using the non-invasive fecal immunochemical occult blood tests (FITs) for detection of fecal hemoglobin (F-Hb) shed from neoplastic lesions, with colonoscopy follow-up for those testing positive, can effectively reduce CRC incidence, severity, and mortality. This is through diagnosing cancers earlier and by detecting and removing the pre-cursor lesions [8]. FIT has a good sensitivity for detection of CRC at 65–87% [9], and the concentration of Hb in the feces is positively associated with risk of advanced neoplasia (advanced adenoma or CRC) [10]. This association has also been observed in individuals with Hb concentrations below the positivity threshold. In the Dutch national screening program, the risk of advanced neoplasia was significantly higher for individuals with fecal Hb concentrations just below the positivity threshold, compared to those returning a fecal Hb of 0 µg Hb/g feces [11]. This was also supported in their follow-up findings that analyzed multiple rounds of screening, where individuals with undetectable and low (< 2 µg Hb/g feces) concentrations of Hb in the feces had the lowest risk for advanced neoplasia [12]. Although these studies were performed in an average risk screening population, the FIT sensitivity for CRC in an elevated risk population is 80% with a specificity of 89% [13]. Taken together, these data suggest that fecal Hb concentrations from FIT completed before surveillance colonoscopy could be a suitable tool to inform postponement of colonoscopies in those who return a very low fecal Hb concentration, thereby reducing the demand on colonoscopy resources.

Optimizing surveillance colonoscopy frequency is challenging, evident by the changing guidelines implemented in Australia for CRC prevention in recent years [14, 15], as well as the different recommendations around the world [16, 17]. While there are studies supporting the benefits of CRC screening [18, 19], the benefits and cost-effectiveness of surveillance colonoscopy are less clear [7], due to limited prospective data. A study using simulation models in the USA suggested that a 10-year colonoscopy interval was more cost-effective than a 5-year interval, whereas an intensive surveillance of 3-yearly intervals was more harmful and resulted in reduced quality-adjusted life years [20]. It may be possible to guide optimal surveillance intervals using quantitative FIT, to balance cancer prevention and overutilization of colonoscopy procedures. When modelling surveillance strategies to compare annual FIT to 5-yearly colonoscopy, FIT was deemed to be as effective and less costly than colonoscopy; it reduced the number of colonoscopies by 45% and was associated with a lower rate of complications [21]. Personalizing surveillance based on FIT Hb concentration would lead to a reduction in the number of colonoscopies. Determining the cost savings of extending surveillance intervals in this context is now needed to justify such changes to clinical care.

Any changes to clinical policy that involves a change to the interval between colonoscopies based on FIT Hb concentration could result in distress amongst consumers. Participants may experience reservations about the efficacy of FIT in detecting abnormalities (i.e., lower response efficacy for FIT than colonoscopy) and have, as a result, greater anxiety and fear of cancer, resulting in decreased quality of life [22]. Alternatively, it may be the colonoscopy procedure itself causing distress. Preference for different surveillance strategies is likely to be, at least in part, dependent on perceived or anticipated participant burden, which influences uptake in CRC screening programs [23]. Perceived inconvenience is a significant predictor of compliance with healthcare recommendations, particularly among some under-served groups in the population [24]. It is therefore possible that individuals at increased risk for CRC will accept changes to surveillance strategies if they perceive the change to be less onerous and as effective. This could also be related to health activation, which describes the skills, confidence, and knowledge a person has in managing their own health [25]. Recent small studies have explored participants’ responses to a possible change to the surveillance process. Participants varied in level of CRC risk but, regardless of risk level, it was reported that an annual FIT was preferred over three yearly colonoscopies in those with no experience of surveillance [26, 27]. Conversely, those with a surveillance history believed that colonoscopy would be more accurate than FIT [26]. This suggests differences in either perceived response efficacy for the two approaches, or a reluctance to change usual practice, or both. It is therefore important to evaluate participant response to actual change in surveillance practice.

The use of FIT Hb concentrations could be a simple and effective way to personalize surveillance colonoscopy frequency. To our knowledge, there have been no randomized controlled trials (RCT) to determine if colonoscopy surveillance intervals can be safely extended in elevated risk CRC surveillance cohorts based on prior low FIT Hb concentration. Based on this, we have designed a randomized controlled trial in a cohort of individuals undergoing regular surveillance colonoscopy, to assess the safety, acceptance, and cost-effectiveness of extending the frequency of colonoscopy based on FIT Hb concentration.

## Objective

This randomized controlled trial will determine if it is safe to delay surveillance colonoscopy procedures by 1 to 2 years in patients with an elevated risk for CRC who have low FIT Hb levels and are undergoing surveillance colonoscopy with a 3- or 5-year surveillance interval. It will also determine the cost savings of delaying colonoscopy procedures, as well as the level of consumer acceptability if such changes were implemented into standard clinical care.

## Methods and design

### Trial design

**Fig. 1:**
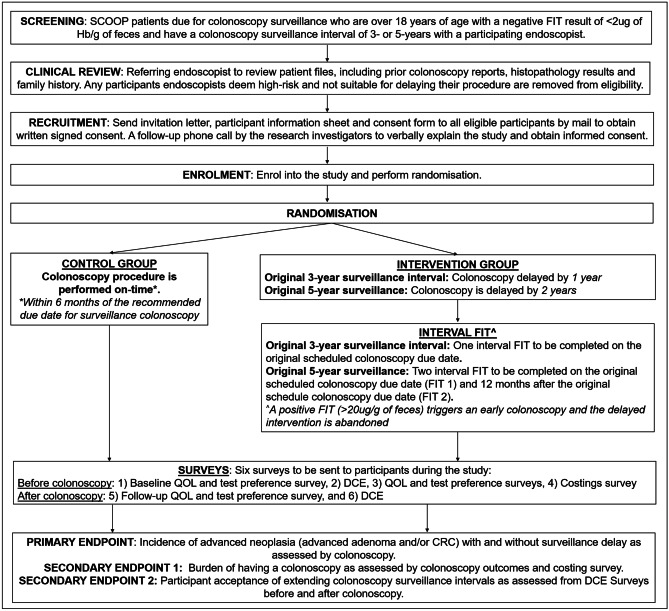
Trial design. Flow chart of participant selection, recruitment, randomization arms, survey participation, and study endpoints. FIT, fecal immunochemical test; Hb, hemoglobin; CRC, colorectal cancer; QOL, quality of life; DCE, discrete choice experiment

This study will be a multicenter, randomized controlled trial at public and private sector hospitals across the southern region of South Australia including Flinders Medical Centre, Noarlunga Hospital, Flinders Private Hospital, and the Tennyson Centre Day Hospital. Invitees will be those at elevated risk for CRC who are enrolled in the Southern Cooperative Program for the Prevention of Colorectal Cancer (SCOOP) program [28, 29] and who are due for surveillance colonoscopy at one of the study sites (Fig. 1). The SCOOP program was established in South Australia in 1999 [29], with the purpose of improving adherence to the Australian National Health and Medical Research council guidelines [15] for frequency of colonoscopy surveillance of individuals with an elevated risk of CRC. Individuals who are eligible for the SCOOP program are those at increased risk of CRC due to any one of the following criteria: (1) a personal history of colonic neoplasia (adenoma, sessile serrated lesion and/or CRC), (2) a family history of a primary degree relative with CRC diagnosed under the age of 55, and/or (3) two primary degree relatives or one primary and one secondary degree relative with CRC diagnosed at any age. As part of the SCOOP, research program individuals are provided with FIT in the interval between their 3- or 5-yearly surveillance colonoscopies [30, 31] at intervals of every 1–2 years. The FIT is offered beginning 1 year after their colonoscopy and 2 years prior to their subsequent scheduled surveillance colonoscopy when on a 5-year surveillance interval.

### Enrolment criteria

Inclusion criteria for enrolment into the study include:

1. Males and females enrolled in the SCOOP surveillance colonoscopy program.
2. Aged older than 18 years of age.
3. Individuals who have either had a previous colonoscopy finding of adenoma, or who have a significant family history of CRC [32].
4. Individuals due for surveillance colonoscopy within the next 6 months after a recommended interval of three or five years.
5. Individuals with a low FIT Hb concentration (< 2 μg Hb/g feces) within the last 3 years of their next scheduled colonoscopy.

Exclusion criteria encompass the following:

1. Individuals with a familial syndrome of CRC or inflammatory bowel disease (IBD).
2. Individuals with a personal history of CRC or sessile serrated lesions.
3. Individuals scheduled for colonoscopy for any indication other than surveillance.
4. Inability to understand study information and give informed consent.

Endoscopist exclusion criteria for this study will be:

1. Not listed as an investigator on the study (for private sector hospital endoscopists).
2. Not performing colonoscopy at a participating hospital.

Individuals with a low FIT Hb result (i.e., < 2 µg of Hb per g of feces) which was completed up to 3 years before their scheduled colonoscopy will be stratified for current colonoscopy interval (i.e., 3 or 5 years). Each group will be randomly assigned (1:1) to intervention or control. Intervention will extend surveillance colonoscopy interval by approximately one third:

- If guideline sets a 3-year surveillance interval (as usually recommended following an advanced adenoma [32]), the interval will be extended to 4 years.
- If guideline sets a 5-year surveillance interval (as usually recommended following a non-advanced adenoma or no neoplasia [32]), the interval will be extended to 7 years.

An interval is considered to have been appropriate if done within 6 months of the set time [33, 34].

Randomization will be performed using the stratified method with block randomization, controlling for (1) the referring endoscopic specialist, (2) the participating private or public hospital, and (3) the recommended surveillance interval. Participants and referring endoscopists will not be blinded to the randomization arm. Those not randomized to the Intervention group will receive standard clinical care, following the Australian National Health and Medical Research Council (NHMRC) guidelines for colonoscopy surveillance intervals [32].

### Study schedule (timing of events)

All individuals from the SCOOP program will be screened for eligibility inclusion and exclusion criteria 6 months before their guideline dictated surveillance colonoscopy. Any individuals that do not respond to study invitation more than 2 months prior to their scheduled surveillance colonoscopy will be excluded from the study. Prior to enrollment, the participant is given a verbal description of the study in detail and are informed that the selection of the study arm is random. Any participant not willing to be assigned to the delegated study arm, either before or after informed consent, will be excluded from the study. If the patient consents to be enrolled and then subsequently withdraws, they will still have their colonoscopy following guideline recommendations.

After obtaining informed consent, all individuals will be sent two surveys (survey 1 and survey 2) by mail prior to them being informed of their randomization (Table 1). Survey 1 will establish baseline quality of life (QOL) using the validated EQ-5D instrument [35] and assess consumer preferences for surveillance strategies and health and psychological predictors of attitude to different protocols. Survey 2 will explore consumer preferences for test attributes using discrete choice experiments (DCE). Upon return of surveys 1 and 2, all participants will be sent a letter by mail informing them of their randomization arm. For those participants who are randomized to the intervention arm, a letter will be sent to their primary health care physician informing them of the change to their patients’ surveillance colonoscopy timing. The QOL survey will be repeated 4 weeks after the participant completes their colonoscopy (survey 3), along with completion of a costings survey (survey 4) within 2 weeks after colonoscopy to determine the costs (direct and indirect) involved with undergoing surveillance (such as time off work, transport costs, support person’s costs, out-of-pocket expenses for colonoscopy, cost of adverse events). Each survey will take less than 20 min to complete. Within the follow-up test preference and QOL survey (survey 5), which is provided one month after colonoscopy, there will also be questions to ascertain satisfaction with the surveillance procedure that they just completed. Finally, a follow-up DCE survey (survey 6) will repeat the DCE survey performed before colonoscopy, once the patients have been informed of pathology findings from their colonoscopy.

**Table 1:** Study schedule listing the timing of events (in months) relative to the guideline recommended colonoscopy due date specified at the time of enrolment

	**Screening/enrolment**	**Survey 1 and survey 2**	**Randomization**	**On-time colonoscopy**	**FIT 1**	**FIT 2**	**Delayed colonoscopy**	**Survey 5**	**Survey 6**
Control	− 6	− 3	− 2	0	N/A	N/A	N/A	7	8
Intervention: 3-year surveillance	− 6	− 3	− 2	N/A	0	N/A	12	13	14
Intervention: 5-year surveillance	− 6	− 3	− 2	N/A	0	12	24	31	32

Two-sample OC-Sensor FIT kits (Eiken Chemical Company, Tokyo, Japan) will be sent annually only to participants who are enrolled into the intervention arm, on their original guideline scheduled surveillance colonoscopy due date. Participants on a 5-year surveillance and with a 2-year delay will receive two FIT kits (1 each year). Participants on a 3-year surveillance will receive one FIT. The colonoscopy procedure will be performed on-time (control group — no FIT required) or on the delayed due date (Intervention group) if the interval FIT is negative (i.e., < 20 µg Hb/g feces). Any participants presenting with symptoms will be scheduled for colonoscopy as soon as possible regardless of trial intervention and will be withdrawn from the study. At the completion of the study and collection of all data, analysis will be performed to determine the primary and secondary outcomes.

### Interval FIT Hb analysis

Fecal Hb analysis in the interval between the delayed colonoscopies for participants randomized to the intervention arm will be performed using two-sample OC-sensor FIT. Testing kits will be provided to participants by mail, with instructions for collection as previously described [36]. Participants will be asked to return the collected fecal samples in the FIT collection devices by mail, within 2 weeks of the first fecal sample being collected. Assessment of fecal Hb concentrations will be performed as per the manufacturer instructions [36]. In the event the FIT result is positive (over 20 µg Hb/g feces or 100 ng/mL of OC-sensor sample buffer in either fecal sample), the surveillance colonoscopy will be scheduled as soon as possible, and the delayed procedure date will be abandoned. These participants will remain enrolled and complete the follow-up survey as dictated in the study schedule.

### Colonoscopy procedure

Colonoscopies will be scheduled within participating institutions across South Australia (Flinders Medical Centre, Noarlunga Hospital, Tennyson Centre Day Hospital, Finders Private Hospital) and will be conducted according to best practice and accreditation requirements at the time. Patients will undergo bowel preparation prior to the procedure (using the preparation and following the instructions specific to each hospital or proceduralist), and the quality of the bowel preparation will be evaluated using the BBPS (Boston Bowel Preparation Scale [37]) with maximum scores of right colon = 3, transverse colon = 3, and left colon = 3, with the total BBPS score equaling 9. Any procedures with a poor bowel preparation, defined as a score of less than 2 in any segment or defined as “poor” in the colonoscopy report, an incomplete intubation, or non-removal of a polyp, will be considered an inadequate incomplete colonoscopy and will be excluded from data analyses. The participant will be scheduled a new procedure date within 6 months of the study colonoscopy procedure date. If the repeat procedure is not completed within 6 months of the study colonoscopy date, the data are excluded from the outcome analysis. The rate of poor bowel preparation (a quantitative BBPS score of less than 2 in any segment, or a qualitative bowel preparation report of “poor”) in the SCOOP program is approximately 5%, which is within the Australian National Health and Medical Research council recommendation of 10% for colonoscopy key performance indicators [15]. If more than 10% of colonoscopy procedures are being excluded due to poor bowel prep, recruitment of participants will continue until the minimum required sample size has been met. The colonoscopy will be performed by trained endoscopists and under anesthesia or sedation. After informed consent is documented, the scope is passed under direct vision with the patient’s pulse and oxygen saturations monitored throughout. The colonoscope is introduced through the anus and advanced to the cecum, which can be identified by the appendiceal orifice and ileocecal valve. Scope withdrawal time is recorded from cecum to rectum. Polyp findings from the colonoscopy are recorded including the Paris classification, location, size, and resection type.

Clinicopathological findings at colonoscopy will be categorized into the following groups: normal, non-neoplastic outcomes, non-advanced adenoma, advanced adenoma, high- or low-risk sessile serrated lesions, or CRC. An advanced adenoma is defined as having any combination of the following features: villous changes, ≥ 10 mm in size, high-grade dysplasia, and traditional serrated adenoma. High-risk sessile serrated lesions are defined as those with dysplasia and/or ≥ 10 mm in size. Non-advanced adenomas and low-risk sessile serrated lesions will be defined as precursor adenomas that do not fit the advanced adenoma or high-risk sessile serrated lesion criteria. Non-neoplastic findings will include hyperplastic polyps, diverticular disease, hemorrhoids, and angiodysplasia, and absence of pathology will be defined as a normal outcome.

### Survey completion

All participants will be mailed paper copies of the surveys for completion, at the timing interval specified in the study schedule (Table 1). Reminder phone calls will be made 4 weeks after the mailing date to ensure compliance with survey completion. Participants are asked to complete the questions to the best of their ability. Survey response will be manually entered into a central computer database.

### Outcome parameters (endpoints)

Primary outcome:

- Incidence of advanced neoplasia (advanced adenoma or CRC) in the control and intervention groups.

Secondary outcomes:

- Incidence of complications from colonoscopy (admissions for bleeding or perforation, or death).
- Risk factors associated with advanced neoplasia including age, sex, family history of CRC, previous history of adenoma, number of prior colonoscopies.
- Cost-effectiveness of a modified surveillance frequency using interval FIT compared to standard surveillance practice.
- Acceptability to consumers of changes to the surveillance strategies and the variables that predict this attitude.

### Sample size

Sample size is based on the judgment that an acceptable upper limit for advanced neoplasia incidence in the intervention arm is approximately 23% (the incidence in cases with a FIT Hb result ≥ 0 µg/g feces [38]). The incidence of advanced neoplasia following a low Hb FIT result in the control group is expected to be approximately 9% [38]. A sample size of 672/group achieves 80% power with an alpha level of 0.05, to detect a 5% difference between the groups using a two-sided *Z* test with pooled variance.

### Statistical analyses

#### Advanced neoplasia incidence

Colonoscopy outcomes of the intervention group will be compared to those from the control group. Incidence of advanced neoplasia (CRC and advanced adenoma), non-significant adenomas, non-neoplastic lesions, and normal outcomes will be compared between groups with univariate (Fisher’s exact and chi-squared test). Odds ratios will be calculated from logistic regression analysis to assess factors associated with the development of advanced neoplasia, adjusted for risk factors associated with neoplasia development such as age, sex, and the original surveillance interval. To account for non-adherence of the timing for the surveillance colonoscopy in the study arm (e.g., colonoscopy was brought forward due to a positive FIT or symptoms), alternative analyses will be incorporated such as per-protocol, modified intention-to-treat, or as-treated, alongside the primary intention-to-treat analysis.

#### Cost analysis

Health economic evaluation will be conducted from both societal and health sector perspectives using a cost-utility analysis (CUA), which involves estimating the incremental costs and effectiveness of extending colonoscopy surveillance intervals based on FIT Hb concentration versus current common practice. The effectiveness will be measured by using quality-adjusted life years (QALYs). An incremental cost-effectiveness ratio (ICER) will be calculated, and results will be plotted on a cost-effectiveness plane. A cost-effectiveness acceptability curve will provide information about the probability that the proposed strategy is cost-effective. The within trial analysis will then be extrapolated using a Markov model, which will consist of health states that are a consequence of the surveillance program, to capture the long-term cost-effectiveness of including FIT within a surveillance program to extend colonoscopy intervals. The lifetime estimates of effect on survival, quality of life, and costs will be estimated from a comprehensive literature review. Both costs and benefits will be discounted at 5% in the base model, in line with government policy.

#### Consumer acceptability

Overall preference for and satisfaction with surveillance type and frequency scenarios will be assessed by direct questions and the DCEs, giving different scenarios of methodology (colonoscopy with or without FIT), frequency, and effectiveness. Themes analyzed include (1) how acceptable they find a modified protocol, (2) how likely they would be to comply with it, and (3) how anxious the changes would make them. The collected demographic and clinical variables will be used to determine if there are any associations with surveillance preferences, and these will include: sex, age, number of previous colonoscopies, number of previous FITs, risk level for CRC (i.e., family history or previous adenoma), time since previous colonoscopy, or FIT. Individual differences in psychological measures will be collected to identify any measures that are likely to predict surveillance preferences: fear of cancer, quality of life, trust in healthcare, perceived convenience of FIT versus colonoscopy, dissatisfaction with previous procedures, relative differences in perceived response efficacy between the two, anxiety about change, and consumer health activation, and self-efficacy for FIT and colonoscopy. Health activation measurement will be with the Consumer Health Activation index (CHAI) [39] which comprises 10 items designed to assess a consumer’s health activation level, where high health activation is positively associated with health self-management [25]. An independent samples *t*-test will compare satisfaction between subjects offered the extended or standard surveillance protocol. A multivariable logistic regression analysis will be applied to determine predictors of preferences for surveillance type and frequency. Independent variables that are not linear on the log-odds scale will be categorized or transformed.

## Discussion

This RCT has been designed to evaluate the efficacy, cost-effectiveness, and consumer acceptability of using FIT to extend colonoscopy surveillance intervals for individuals who are considered at elevated risk of CRC. The design process was implemented to minimize introduction of bias and to ensure the safety of all participants involved. To the best of our knowledge, this is the first RCT of its kind to utilize the FIT to personalize surveillance intervals in an elevated risk cohort.

The primary outcome of this study will be to assess incidence of advanced neoplasia in those individuals with a low FIT Hb concentration who have an on-time colonoscopy dictated by current guidelines versus those with a delayed colonoscopy procedure. Prospective studies in average and elevated risk cohorts have shown that the incidence of advanced neoplasia in those with low or undetectable fecal Hb are low. A study by Digby et al. showed that participants with a personal history of adenoma or a family of CRC (elevated CRC risk) who returned a fecal Hb level ≤ 2 µg Hb/g had a 7.3% rate of advanced neoplasia at colonoscopy, and for those with undetectable fecal Hb (0 Hb/g), the rate was only 2.8%, which is equivalent to an overall relative risk reduction of 59.4% [40]. Senore et al. [41] demonstrated that in a population-wide screening program of average-risk individuals, a combined undetectable fecal Hb from multiple previous rounds of FIT (about 50% of the participants) led to a 1.4% risk of finding an advanced neoplasia over the subsequent two rounds of colonoscopy compared to those with a FIT Hb level ≥ 20 µg/g (about 0.7% of the population) who had an 18-fold increase in their cumulative advanced neoplasia risk over the same time interval [41]. The Dutch CRC screening work group conducted a large-scale prognostic model that used Hb concentrations from two biennial FIT rounds to predict colonoscopy outcomes (advanced neoplasia) after a third round of a positive FIT (≥ 47 µg Hb/g feces) [12]. Their data showed that only 1.8% of individuals with low Hb in the feces in the first FIT round were diagnosed with advanced neoplasia after a third round positive FIT, and only 3.9% were diagnosed with advanced neoplasia after a low Hb at FIT round 2. Collectively, these data report a low risk for advanced neoplasia in individuals with a low FIT Hb concentration, which supports the plan to extend surveillance colonoscopy intervals in individuals with low fecal Hb. However, a limitation of these studies was that outcomes were only measured in those that eventually had a positive FIT with colonoscopy follow-up or in those incidentally diagnosed with interval cancer. Those with a negative FIT result were not followed up with colonoscopy to determine the actual incidence of advanced neoplasia. RCTs are now needed to validate these findings in an unbiased manner to provide evidence to support changing current surveillance colonoscopy guidelines.

While assessing the risk of developing advanced neoplasia is crucial to the outcome of this study, one of the secondary outcomes from this study will be to determine what costs might be saved with the proposed changes to surveillance guidelines. In Australia, the use of colonoscopy has been rising for over two decades [42], but the capacity for this costly and invasive procedure in a strained public healthcare system is limited. The impact that colonoscopy has on the environment is also significant, in part due to the sheer case load of patients every year, carbon heavy travel required for patients and their support person to attend appointments, various waste streams that use copious amounts of water, high consumption of single-use consumables, and resource-heavy decontamination processes [43]. The procedure also requires a time commitment of at least 3 days away from normal activities to allow the completion of bowel preparation the day before, sedation/anesthesia on the day of the procedure, and followed by a recovery day. This time away from normal duties can have a significant impact on economic productivity due to the required absences. An RCT that demonstrates the actual cost savings in reducing the number of colonoscopies each year, while minimizing the impact of incidence of advanced colorectal neoplasia, will provide the evidence needed to support implementing changes to policy guidelines on CRC surveillance.

Another key secondary outcome of the planned study will investigate preferences for surveillance strategies, as well as the associated influencing variables within a large cohort from the SCOOP program, in order to identify whether the proposed changes to colonoscopy surveillance guidelines using FIT are acceptable. The strength in surveying this group is that they have undergone surveillance colonoscopy as well as being offered interval screening with FIT as part of the SCOOP program already. Earlier studies have shown that the regardless of an individual’s risk for developing CRC (i.e. average or elevated), a FIT offered to participants annually was preferred over a 3-yearly colonoscopy in individuals who had never undergone surveillance colonoscopy [26, 27]. However, those individuals who had already participated in CRC surveillance colonoscopy believed that colonoscopy would be more accurate than FIT [26]. These studies suggest that there are not only differences in response efficacy (i.e., whether they believe the recommended surveillance strategy will detect the neoplasia) between colonoscopy and FIT, but that there may be hesitancy for individuals to change standard practice based on these beliefs. The reported RCT study design presented here will provide the evidence needed to answer these questions in an unbiased approach.

There are some limitations to this study. There is uncertainty around consent and participation rates in a cohort of individuals who are already aware that they are at above-average risk for developing CRC and whom have already been informed by their primary care physicians and proceduralists that they are required to perform these colonoscopies regularly and on time. This may lead to only individuals who are personally comfortable to delay their colonoscopy procedure enrolling in the study, thereby creating a bias in the population. Furthermore, potential reluctance of eligible participants in delaying their procedure could also lead to lower than anticipated sample size, which may impact data analysis. Finally, although this study will be limited to an Australian population at elevated risk of CRC due to a personal history of adenomas and/or a significant family history of CRC, findings could be interpreted to support implementing the same strategy to those who undergo colonoscopy only and are at average risk for CRC, as is the case in the USA.

In conclusion, we report the study design of an RCT that is expected to determine the efficacy, cost-effectiveness, and consumer acceptability of personalizing colonoscopy surveillance intervals using FIT. The results from this study will create an evidence-base for recommendations that would be implementable in clinical practice and practice changing with national guidelines. Such outcomes have the capacity to reduce the burden placed on hospital resources and provide an overall improved patient experience, without increasing the incidence rate of advanced colorectal neoplasia.

## Data Availability

Data is available upon request.
